# The Leicester Provident Dispensary

**Published:** 1888-05-19

**Authors:** M. Maxfield

**Affiliations:** Vice-Chairman of the Board of Governors.


### The Editor's Letter-Box.

[Our Correspondents are reminded that prolixity is a great bar to publication, and that brevity of style and conciseness of statement greatly facilitate early insertion.]

### THE LEICESTER PROVIDENT DISPENSARY.

Sir,—In your number of April 7th, you state in relation to the Leicester Provident Dispensary that "the Rev. A. A. Isaacs has brought some very serious charges against the management, and demands an inquiry, which the governors decline to grant." In your number of April 28th you insert a resolution passed at a meeting of the members referring the matter to his Worship the Mayor. I beg to say that the Board of Governors, having nothing to conceal, have no hesitation in leaving the settlement of the dispute in his hands, with one reservation, that the committee of inquiry shall not be composed of members of the medical profession who are in no way connected with the dispensary, and who have always looked upon its work as opposed to their interests. You also state "that twenty-six of the medical men connected with the institution have signed a memorial stating that essential changes are necessary if the usefulness of the institution is to be maintained." Allow me to say that twenty-two of these medical men are neither members of the medical staff of the dispensary, nor are they even to be found on the list of its subscribers. The dispensary has been in existence as a provident institution over a quarter of a century, and has over thirty-five thousand members. No greater proof of its successful working and its acceptance by the working population need be adduced. M. Maxfield,

Vice-Chairman of the Board of Governors.

May 11th, 1888.

